# Supplementary material for: Divergent Effects of Acute and Prolonged Interleukin 33 Exposure on Mast Cell IgE-Mediated Functions
Source: Front Immunol. 2019 Jun 19;10:1361. doi: 10.3389/fimmu.2019.01361 (PMC6593472; doi:10.3389/fimmu.2019.01361)
Supplement: Supplementary file 1 [file Data_Sheet_1.PDF]

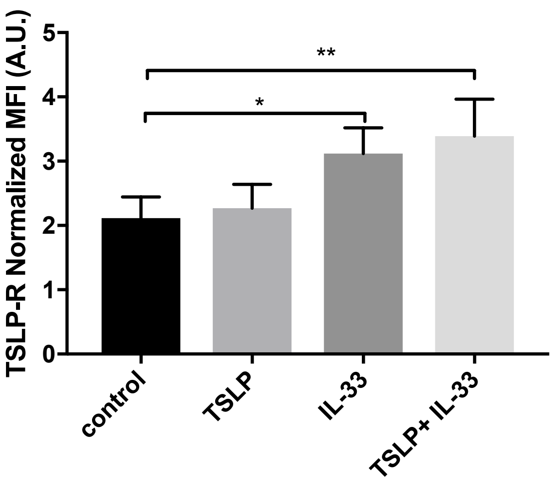

Supplementary Figure 1.
ROSA cells were repeatedly treated every day for four days with 10 ng/ml IL-33, TSLP or a combination of both; thereafter, the levels of the TSLP-R was measured on fixed and permeabilized cells, staining both surface and intracellular TSLP-R. Data shown were pooled from 2 independent experiments, n=4.

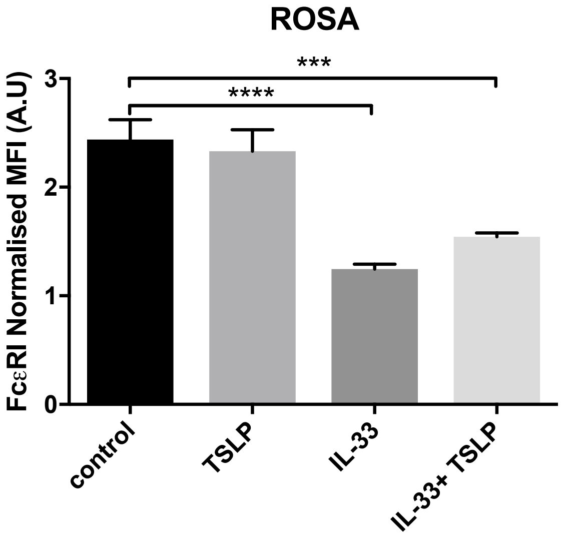

Supplementary Figure 2.
ROSA cells were repeatedly treated every day for four days with 10 ng/ml IL-33, TSLP or a combination of both without the addition of IL-4. Surface FcRI receptor expression was measured by flow cytometry. The MFI of FcRI was normalized to that of the isotype control. Data shown were pooled from 3 independent experiments, n=6.
